# The Southern Journal of the Medical and Physical Sciences

**Published:** 1857-04

**Authors:** 


### "THE SOUTHERN JOURNAL OF THE MEDICAL AND PHYSICAL SCIENCES."

We are pleased to note the fact, that this Journal is not "*dead*" but has only been in a state of suspended animation, and that it has reappeared with new life and vigor. We can commend it as an excellent Journal and deserving success, which we hope it will receive in full measure.

The Journal is published in Knoxville, Tennessee, contains eighty pages—monthly, at $3 a year, and edited by Richard O. Currey, A. M., M. D.,—evidently a gentleman of talent and energy.

